# A Sizable Aortic Root Paravalvular Mycotic Pseudoaneurysm

**DOI:** 10.1155/2016/7924631

**Published:** 2016-10-11

**Authors:** Ahmad Saeed Azhar, Noran M. Abu-Ouf

**Affiliations:** Department of Pediatrics, Faculty of Medicine, King Abdulaziz University, Jeddah, Saudi Arabia

## Abstract

Mycotic aneurysm is an established condition first identified in 1885 by Sir William Osler. It is linked to malignant endocarditis. With prevalence of 0.7–2.6% of all cases of aortic aneurysms, it is associated with a significant rate of mortality and morbidity. Physicians should be highly cautious, as diagnosis and effective treatment for this condition are difficult. The following is a case report of a 13-year-old pediatric patient diagnosed with mycotic aneurysm. Before an adequate treatment plan could be developed and implemented, patient's status worsened swiftly and was ultimately terminal. This case is the sole instance of this condition evidenced in the last ten years in Saudi Arabia.

## 1. Case Report

A 13-year-old, not known to have had any previous medical or surgical problems, presented to the Emergency Department (ER) with a two-week history of fever, shortness of breath, a dry cough, chills, and night sweats. One week prior to presentation, he had experienced orthopnea, palpitations, and central chest pain. On initial physical examination, the patient blood pressure was 110/50 mmHg, and he was tachycardic (147 beats/min.), febrile (39°C), and tachypneic (55 breath/min). Oxygen saturation (97%) was enabled by use of a 10 L facial mask. A chest examination revealed scattered crepitation and a gallop rhythm with pansystolic and early diastolic murmurs (grade 4/6 with thrill). An abdominal examination revealed a tender liver; however, there was no palpable mass or organomegaly. Blood analysis revealed leukocytosis (white blood cell count, 16.3/*μ*L) with a heightened erythrocyte sedimentation rate of 68 mm/H and a C-reactive protein concentration of 98 mg/L. Further, all cultures (urine, blood, sputum, and throat viral cultures) were negative after 5 days of incubation. Common laboratory testing of urine, electrolytes, and renal and hepatic profiles was normal. Plain radiography of the chest was also found to be normal.

**Figure 1:**
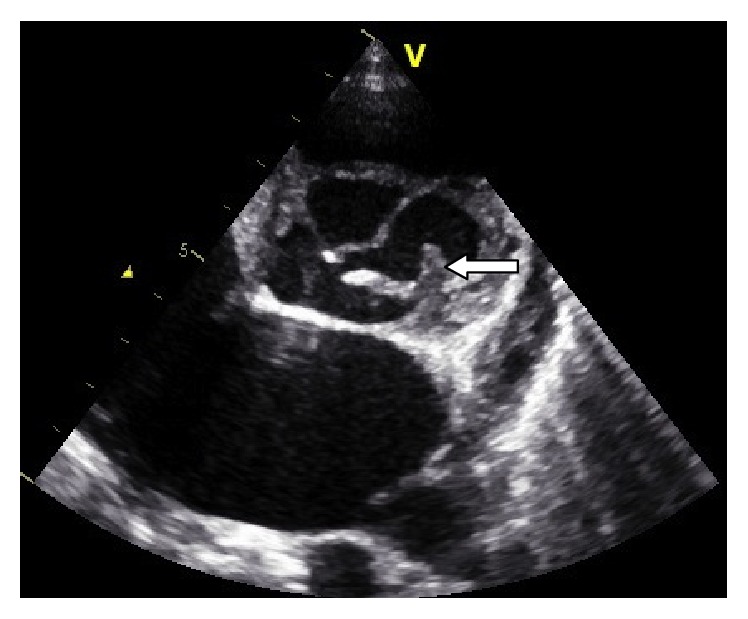
Modified short access view at the level of the aortic root showing large mycotic pseudoaneurysm.

Initially, the patient was admitted from the ER to the pediatric medical ward. Later at the same day, when he showed signs of respiratory distress, he was transferred to the pediatric intensive care unit (PICU) for further management. In the PICU, the patient was kept on bilevel positive airway pressure and steroids. Consequently, the patient was started on Furosemide (20 mg/8 h), dobutamine (10 mcg/kg/min), and antibiotics; ceftriaxone was started immediately in the ER. Later, at the same day, broad-spectrum antibiotics were added (Vancomycin and Gentamycin) as a treatment of bacterial endocarditis, until any result of blood culture and sensitivity testing is established. All medications were given intravenously. An echocardiography was performed immediately, which indicated evidence of heart failure and infective endocarditis, with suspected mycotic aortic aneurysm (Figure 1). There was mild to moderate aortic valve regurgitation and fair myocardiac contractility with an Ejection Fraction (EF) of 42% and Shortening Fraction (SF) of 18%.

**Figure 2:**
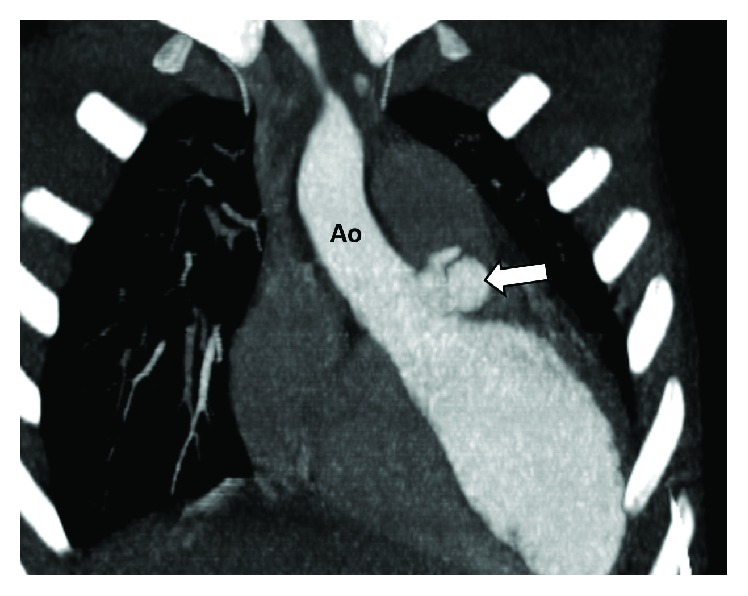
Coronal maximum intensity projection of the aorta (Ao) demonstrating a paravalvular mycotic pseudoaneurysm arising from the aortic root (arrow).

**Figure 3:**
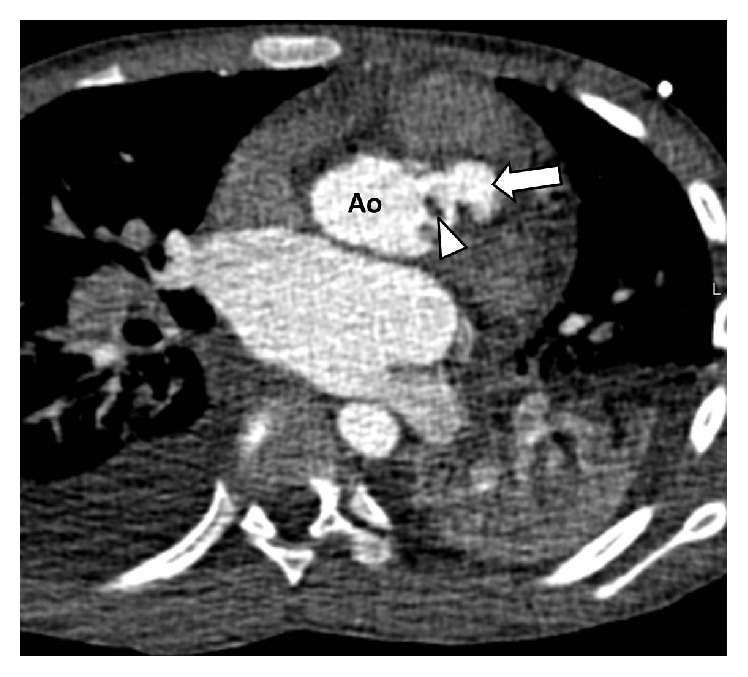
Coronal maximum intensity projection computed tomography of the aorta (Ao) showing a paravalvular mycotic pseudoaneurysm stemming from the aortic root (arrow).

Urgent electrocardiogram- (ECG-) synchronized intravenous (IV) contrast-enhanced multidetector computed tomography (MDCT) imaging of the heart and aorta was subsequently performed, demonstrating a sizable aortic root paravalvular mycotic pseudoaneurysm with internal vegetation (Figures 2 and 3). Accordingly, the diagnosis of both mycotic aortic aneurysm and heart failure due to infective endocarditis was confirmed.

The patient was scheduled for surgical intervention on the following day (aortic aneurysm excision and graft replacement), with a plan of cleaning and debridement of the aortic valve and root, followed by reconstruction with aortic homograft. Unfortunately, the patient developed arrhythmia, hypotension, and shock hours later, with subsequent desaturation and cardiac arrest (≤2 minutes). Cardiopulmonary resuscitation (CPR) was applied for 20 minutes, but there was no response and the patient died. Autopsy was intended to be taken in order to confirm the diagnosis, but, unfortunately, parents refused it.

## 2. Discussion

Mycotic aneurysm of the aorta, especially with a thoracic element, rarely occurs in children and it can result in mortality subsequent to intervention delay [1, 2]. When it does occur, it is usually in children with such underlying congenital heart diseases as persistent ductus arteriosus, aortic coarctation, or aortic valve irregularities [3]. In certain instances, it may evolve as a consequence of surgical trauma after an aortotomy or manipulation of a catheter within the artery [3]. It is thought that there are three main causes. First, it may be a result of infected emboli following infective endocarditis or systemic bacteremia, which gather in a diseased artery, triggering damage to the wall. This ultimately can result in an infected aneurysm. A second scenario is that it may stem from an extravascular infectious focus, which leads to necrosis in the arterial wall and ensuing aneurysm development. A third scenario is superinfection of an already established aneurysm [4, 5]. Depending on the extent of arterial wall infection, mycotic aneurysms may be separated into true aneurysms, which occur when all three strata of the arterial wall are compromised, or false aneurysms (also referred to as pseudoaneurysms), which can occur when aneurysmal dilation includes part of the media, the adventitia, and periadventitial tissues [6]. Clinical presentation of this condition varies widely and is contingent on the affected aspect of the aorta. Usually, at presentation, patients are septic and febrile [7]. Prompt diagnosis and proper management with antibiotics accompanied by pressing surgical intervention are vital to optimizing patient's status [7]. Nevertheless, mycotic aortic aneurysms continue to be linked to an elevated risk of adverse health sequelae and high mortality rate, irrespective of improvements in vascular surgery [8]. Prevalent outcomes from mycotic aneurysm that do not receive treatment comprise rupture, fistula formation, sepsis, erosion, and fatality [4, 7].

In this case, diagnosis of infective endocarditis was delayed until patient's clinical presentation. It is possible that the patient had a previously undiagnosed congenital heart condition, leading to infective endocarditis and ultimately the development of a mycotic aortic aneurysm. To confirm this diagnosis, we utilized a noninvasive ECG-synchronized IV contrast-enhanced cardiac MDCT, which has been shown to be efficacious in assessing infective endocarditis [9], especially for detecting the degree of paravalvular extension within the infection [9]. Further, it is effective in determining coronary artery status, thus supporting the avoidance of high-risk preprocedural invasive catheter coronary angiography [10, 11].

Even though the patient received antibiotic and anticardiac failure treatment as soon as the diagnosis was suspected, it seems that patient's situation was deteriorating, which may have been avoided if the root cause had been determined earlier. Despite no clear established cause of mortality, it is considered probable that the patient experienced a ruptured mycotic aortic aneurysm, resulting in hypovolemic shock, as indicated by desaturation, hypotension, and cardiac arrest. Another possible cause, such as sepsis, was eliminated, as the patient was taking appropriate antibiotic medication. Unfortunately, autopsy has not been done which could have confirmed findings on imaging and confirmed the definite cause of death (probably rupture, but coronary embolism from a vegetation is also an option); however, parents refused it.

## 3. Conclusion 

This case emphasizes the critical importance of prompt diagnosis followed by rapid surgical treatment in patients with mycotic aortic aneurysm in order to avoid patient mortality. Research indicates that the standard approach for mycotic aneurysm is rapid surgical treatment accompanied by longitudinal antibiotic treatment in order to limit widespread infection and to accomplish cardiovascular stability. The most important message from this case is not to delay diagnosis and high-risk surgery, which all need to be done simultaneously in the same day at least instead of doing things step-by-step to prevent rupture and mortality.
